# Supplementary material for: The protected physiological state of intracellular Salmonella enterica persisters reduces host cell-imposed stress
Source: Commun Biol. 2021 May 4;4:520. doi: 10.1038/s42003-021-02049-6 (PMC8096953; doi:10.1038/s42003-021-02049-6)
Supplement: Supplementary file 2 — Supplementary Information [file 42003_2021_2049_MOESM2_ESM.pdf]

Supplementary Information

Supplementary Tables

Supplementary Table S 1. Plasmids used in this study

Plasmid	Relevant genotype*	Source/reference
p5204	P_{EM7}::*dsred* P_{cypD}::*sfgfp* (frameshift)	[1]
pWRG167	P_{EM7}::*sfgfp*	[2]
p4928	P_{EM7}::*tagrfp-T* P_{tetA}::*sfgfp*	[3]
p5055	P_{EM7}::*dsred* P_{htrA}::*sfgfp*	[3]
p5084	P_{EM7}::*dsred* P_{msrA}::*sfgfp*	[1]
p5085	P_{EM7}::*dsred* P_{trxA}::*sfgfp*	[1]
p5205	P_{tetA}::*dsred* P_{msrA}::*sfgfp*	This study
p5300	P_{tetA}::*dsred* P_{htrA}::*sfgfp*	This study
p5302	P_{tetA}::*dsred* P_{trxA}::*sfgfp*	This study
p5426	P_{BAD}::*dsred* P_{tetA}::*sfgfp*	This study
p5418	P_{tetA}::*dsred* P_{cypD}::*sfgfp* (frameshift)	This study
p5419	P_{BAD}::*tag-rfp-T* P_{tetA}::*sfgfp*	This study

* all plasmids confer resistance to carbenicillin

Supplementary Table S 2. *Salmonella enterica serovar* Typhimurium strains used in this study

Strain	Relevant characteristics	Source/reference
NCTC12023	wild type	NCTC, lab stock
MvP1890	Δ*ssaV*::FRT	[1]
MvP1980	Δ*sseF*::FRT	[1]
MvP503	Δ*sifA*::FRT	[4]
MvP2600	Δ*dksA*::FRT	[3]

Supplementary Table S 3. Oligonucleotides used in this study

Designation	Sequence 5'-3'
for Gibson assembly cloning	
Vf-p5084 (2)	AAGGAGATGGCGCCCAACAGTC
Vr-p5084 (2)	CGGGAAAGAGGAGAAAGTATGGC

1f-p5084-PtetA (2)	GTTGGGCGCCATCTCCTTTGCTTTTAAGACCCACTT
1r-PtetA-p5084 (2)	CATACTTTTCTCCTCTTTCCCGTTCACTTTTCTCTATCACTGA
Vf-p4928	GGCCGCAGAGAATATAAAAAGC
Vr-p4928	CGATATCGGTACCGGGAAAGAG
1f-p4928-Para	GCTTTTTATATTCTCTGCGGCCATCGATGCATAATGTGCCTGTC
1r-Para-p4928	CTTTCCCGGTACCGATATCGACCATGGTGAATTCCTCCT
Vf-sfGFP	ATGCGCAAAGGCGAAGAACTGT
Vf-pMW211-V2	ATGGCATCCACCGAGGAC
1f-p5084-PBAD	GTCCTCGGTGGATGCCATACTTTTCTCCTCTTTCCC
1r-tetA-p5084	GTTCTTCGCCTTTGCGCATGATATCCTCCTCTTGCCATCT
Check primer	
PtetA-p5084-Check-For	TCGCCCTCGATCTCGAACTC
PtetA-p5084-Check-Rev	GAATAAGAAGGCTGGCTCTG
Para-p4928 Check-For	CCGACCGCTGGGAATGAAAG
Para-p4928 Check-Rev	AAGGTGCAGAGCCAGCCTTC
dsred-p5419 Check-For	GCACTTTGAAGCGCATGAAC
dsred-p5419 Check-Rev	GGTCGGCAAACAAATTCTCG

Supplementary Figures and Figure Legends

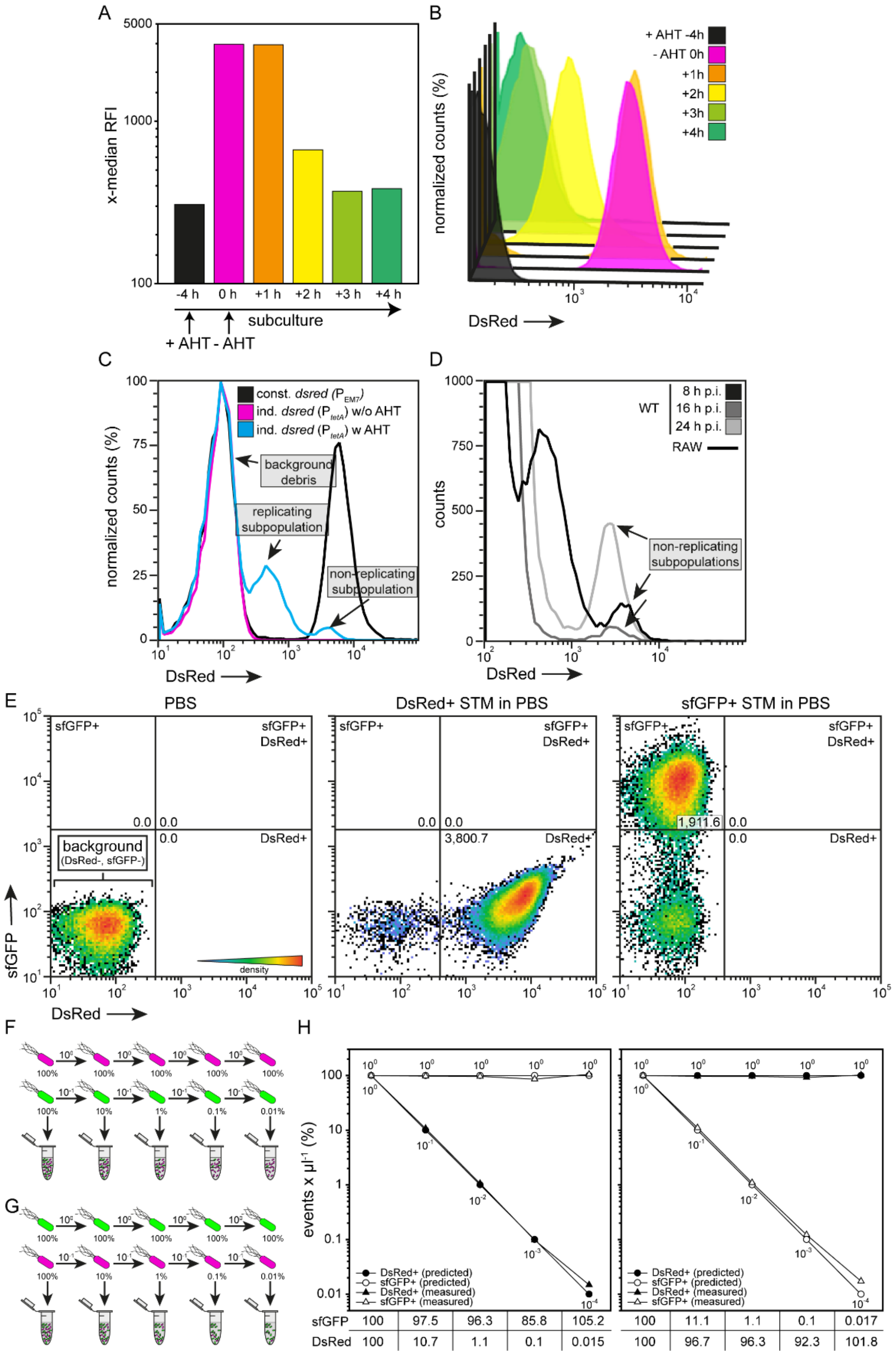

Supplementary Fig. S 1: A dual-fluorescence reporter suitable to detect the non-replicating subpopulation of intracellular STM. A) STM WT harboring dual-fluorescence reporter with AHT-inducible *dsred* expression and *msrA*::*sfgfp* was grown in LB medium o/n and diluted in fresh LB medium containing AHT for further subculture. After 4 h of subculture, AHT was removed by centrifugation and washing. Subsequently, bacteria were subcultured in fresh LB without AHT. At

indicated time points samples were taken, fixed and subjected to FC. The x-median represents the AHT-induced DsRed intensity of the entire bacterial population, and the respective histograms (B). C) STM WT harboring AHT-inducible or constitutive dual-fluorescence reporter for *msrA* was grown o/n in LB medium in the presence of AHT if necessary. Before infection, AHT was removed. RAW264.7 macrophages were infected, and lysed 8 h p.i. Liberated STM were recovered, fixed and subjected to FC. As negative control, AHT was omitted from o/n cultures. The histogram shows that intracellular NR STM can be detected inside RAW264.7 macrophages showing the same DsRed intensity compared to intracellular STM WT harboring the constitutive dual-fluorescence reporter for *msrA*. If AHT was omitted, no DsRed-positive STM can be detected. D) NR subpopulations of intracellular STM WT can be detected after various time points post infection. The same experiments were performed for the AHT-inducible dual-fluorescence reporter for *trxA* and *htrA,* with the same outcome. E-H) Detection accuracy of bacterial particles on an Attune NxT cytometer. STM WT strains constitutively expressing *dsred* or *sfgfp* were grown in LB medium o/n. E) Controls of PBS without STM, only DsRed-positive STM, or only sfGFP-positive STM are shown. Events per μl are indicated in each gate. Then, mixed ratios of DsRed- and sfGFP-expressing STM were prepared in PBS. Either a constant high amount of sfGFP-positive STM mixed with an equal, 10, 100, 1,000, or 10,000-fold reduced amount of DsRed-positive STM (F), or a vice versa (G) was prepared and directly subjected to FC. H) The relative amount (events x μl^{-1}) of sfGFP- and DsRed-positive STM measured in the first sample was set to 100% ($10^0/10^0$). Mixtures with gradual 10-fold reduction of red or green fluorescent STM within a sample containing constant high amount of green or red fluorescent STM, respectively, were quantified. The measured value (events x μl^{-1} in %) compared to the first sample containing high amounts of both, red and green fluorescent STM, is indicated below.

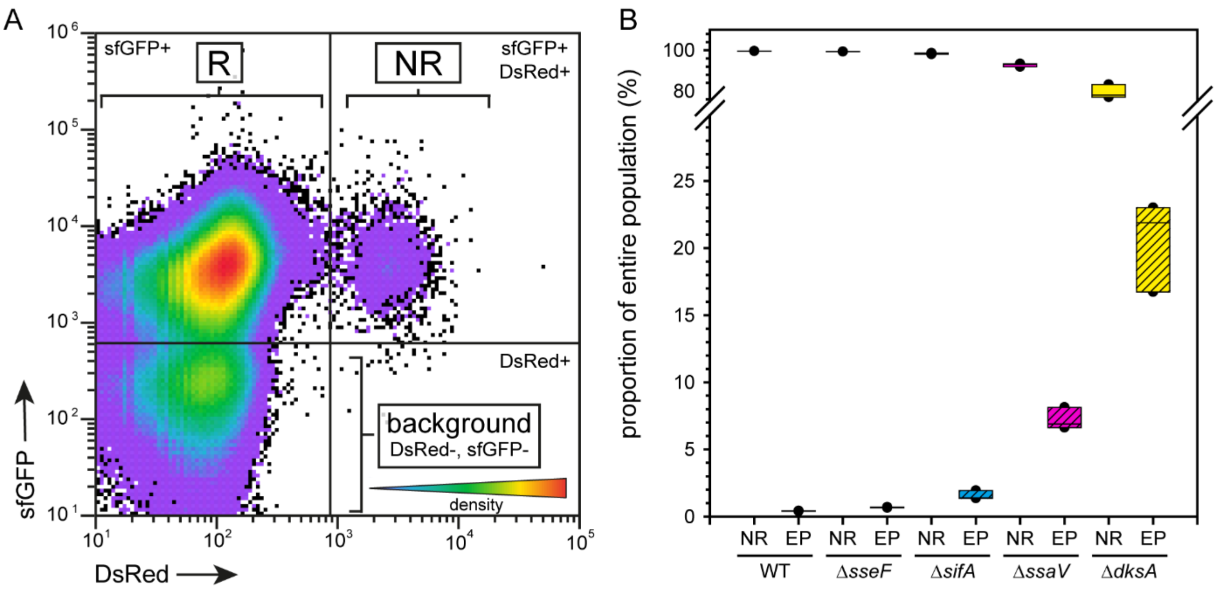

Supplementary Fig. S 2: A minor fraction of the intracellular population consists of non-replicating STM. STM WT, Δ*sseF*, Δ*sifA*, Δ*ssaV* and Δ*dksA* harboring AHT-inducible dual-fluorescence reporter for *msrA* were grown o/n in LB medium in the presence of AHT. AHT was removed prior to infection. RAW264.7 macrophages were infected and lysed 24 h p.i. STM released from host cells were recovered, fixed and subjected to FC. A) Analyses of the entire intracellular bacterial population for AHT-induced DsRed and *msrA*-induced sfGFP intensity shows three different populations. DsRed-negative (DsRed-) and sfGFP-negative (sfGFP-) events represent the background signal consisting of host cell debris. DsRed- and sfGFP+ events represent the replicating (R) subpopulation of intracellular STM and DsRed+ and sfGFP+ events represent the non-replicating (NR) subpopulation. B) Gating on, and quantification of R and NR subpopulations indicated the small size of the population of NR intracellular STM WT.

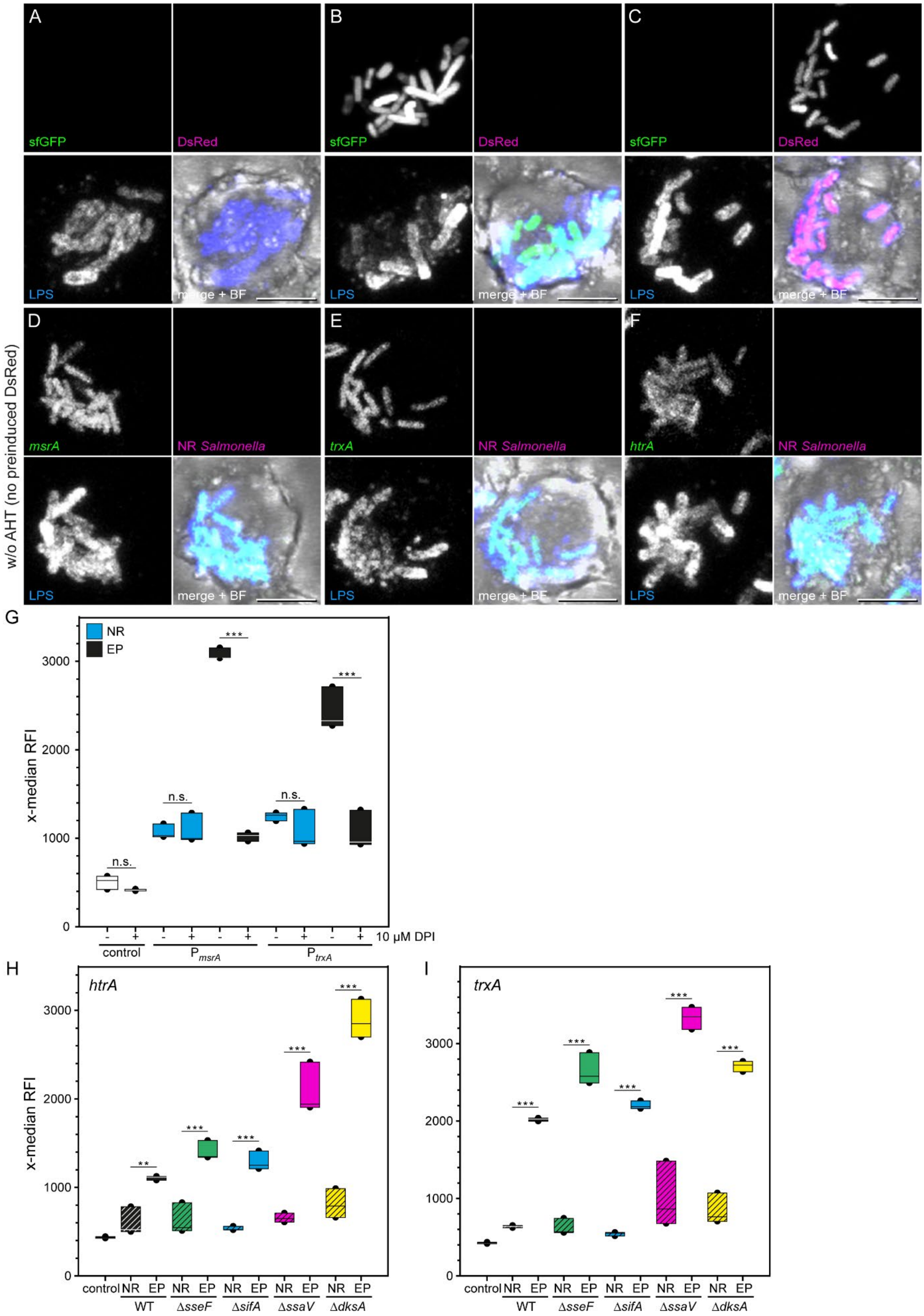

Supplementary Fig. S 3: Functional characterization of dual fluorescence reporters. A-F) STM WT harboring AHT-inducible (NR) dual-fluorescence reporters for *msrA, trxA,* or *htrA* were grown o/n in LB medium without AHT. RAW264.7 macrophages were infected and fixed 8 h p.i. for

fluorescence microscopy. STM were immuno-stained against O antigen (blue). As fluorescence controls, STM WT without expression of any FP (A), STM WT constitutively expressing *sfgfp* (B), and STM WT constitutively expressing *dsred* (C) were used. Induction of P_{msrA} (D), P_{trxA} (E), or P_{htrA} (F) is shown in green. NR STM were not detected due to absence of AHT in o/n culture. Representative cells are shown. Scale bars, 5 µm. G) STM WT harboring AHT-inducible (NR), or constitutive (EP) dual-fluorescence reporters for *msrA* or *trxA* were grown o/n in LB medium. As negative control, a dual FP reporter plasmid with *sfgfp* inactivated by a frameshift was used (control). AHT was present for AHT-inducible reporters and removed before infection. RAW264.7 macrophages were infected and 10 µM diphenyleneiodonium chloride (DPI) was added at 1 h p.i. if indicated. Host cells were lysed 8 h p.i., liberated STM were recovered, fixed, and subjected to FC analyses. The x-median represents the P_{msrA}- or P_{trxA}-induced sfGFP signal of the DsRed-positive intracellular bacterial population at 8 h p.i. Means and standard deviations of a representative experiment are shown. H, I) STM WT, Δ*sseF*, Δ*sifA*, Δ*ssaV,* and Δ*dksA* strains harboring AHT-inducible (NR), or constitutive (EP) dual-fluorescence reporter for *htrA* (H) or *trxA* (I) were grown o/n in LB medium. AHT was present for AHT-inducible reporters and removed before infection. RAW264.7 macrophages were infected, lysed 24 h p.i., liberated STM were recovered, fixed, and subjected to FC analyses. As negative control, a dual FP reporter plasmid with *sfgfp* inactivated by a frameshift was used. The x-median represents the *htrA*- or *trxA*-induced sfGFP signal of the DsRed-positive intracellular bacterial population at 24 h p.i. Means and standard deviations of a representative experiment are shown.

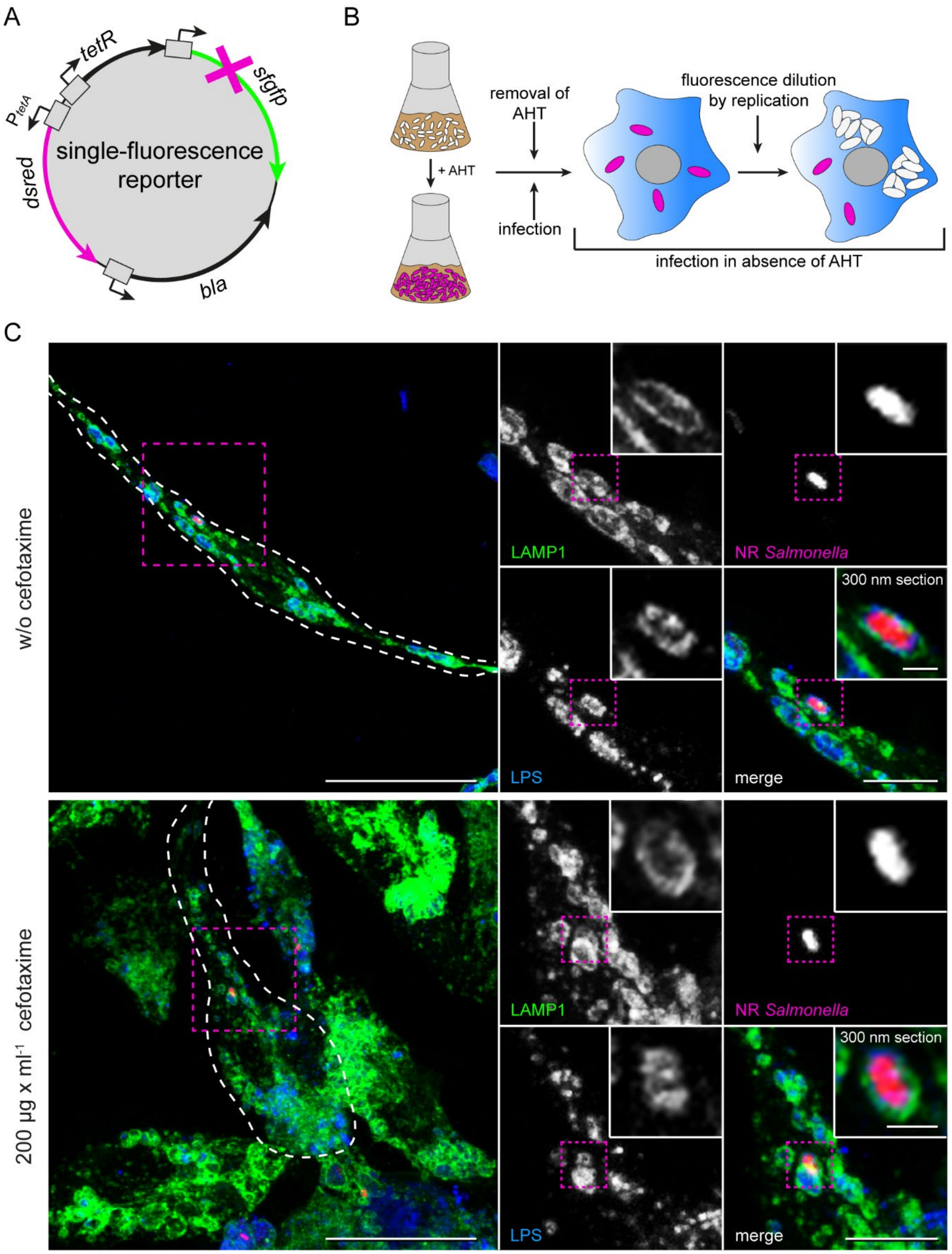

Supplementary Fig. S 4: Non-replicating persisters reside inside SCV in infected host cells. A) For the analysis of NR intracellular STM, the EM7 promoter of the non-induced *sfgfp* frameshift control plasmid was replaced by the tet-ON cassette to experimentally induce expression of *dsred* by AHT. B) After addition of AHT to growing STM cultures, bacteria synthesized DsRed. Prior infection, AHT was removed by centrifugation and washing and infection was performed without addition of AHT. Intracellular R STM lose DsRed via fluorescence dilution, while intracellular NR

STM maintain DsRed level and were detected as DsRed-positive STM. C) STM WT harboring AHT-inducible single FP reporter was grown o/n in LB medium in the presence of AHT. Before infection, AHT was removed. RAW264.7 LAMP1-GFP macrophages were infected, 10 h p.i. cefotaxime was added to the cells if indicated and fixed 24 h p.i. for fluorescence microscopy. Fixed intracellular STM were immuno-stained against O antigen (blue). LAMP1 is shown in green, Red fluorescence (displayed magenta) was present or absent in NR STM or R STM, respectively. Representative cells are shown. Scale bars, 20, 5, 1 µm in overview, details, and zoom-in, respectively.

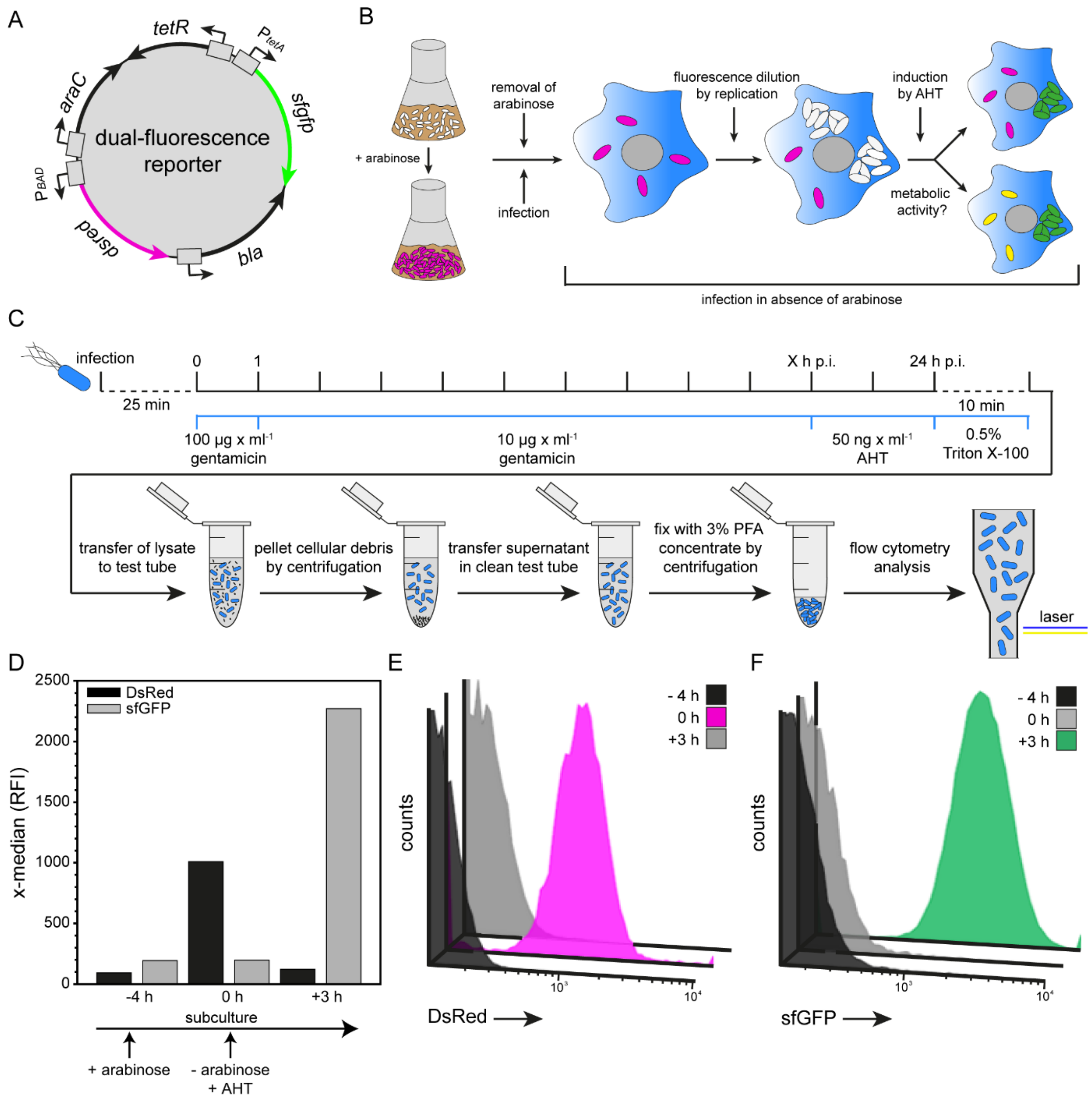

Supplementary Fig. S 5: Dual-fluorescence reporters for analyses of metabolic activity of intracellular NR STM. A) For the measurement of metabolic activity of NR intracellular STM the EM7 promoter and the *msrA* promoter of the dual-fluorescence reporter for *msrA* was replaced by the arabinose-inducible promoter cassette [5], and the tet-ON cassette to be able to artificially induce the expression of *dsred* by arabinose, and *sfgfp* by AHT. B) By addition of arabinose to a growing STM culture, bacteria synthesized DsRed. Prior infection, arabinose was removed by centrifugation and washing and Infection was performed in absence of arabinose. intracellular R STM lose DsRed via fluorescence dilution, while intracellular NR STM maintain DsRed and were detected as DsRed-positive STM. In addition, the metabolic activity was determined by addition of AHT leading to sfGFP synthesis by metabolically active bacteria. C) RAW264.7 macrophages were infected and non-phagocytosed STM were killed by gentamicin. At indicated time points AHT was added and host cells were lysed 24 h p.i. After removal of cell debris, liberated STM were fixed and subjected

to FC. D-F) STM WT harboring double-inducible dual-fluorescence reporter was grown in LB medium o/n and diluted in fresh LB medium containing arabinose for further subculture (-4 h). After subculture for 4 h, arabinose was removed by centrifugation and washing (0 h). Subsequently, bacteria were further subcultured for 3 h in fresh LB without arabinose, but containing AHT (+3 h). Samples were collected, fixed and subjected to FC at indicated time points. D) The x-median represents the arabinose-/AHT-induced DsRed/sfGFP intensity of the entire bacterial population. Respective histograms for DsRed intensity (E), and sfGFP intensity (F) are shown.

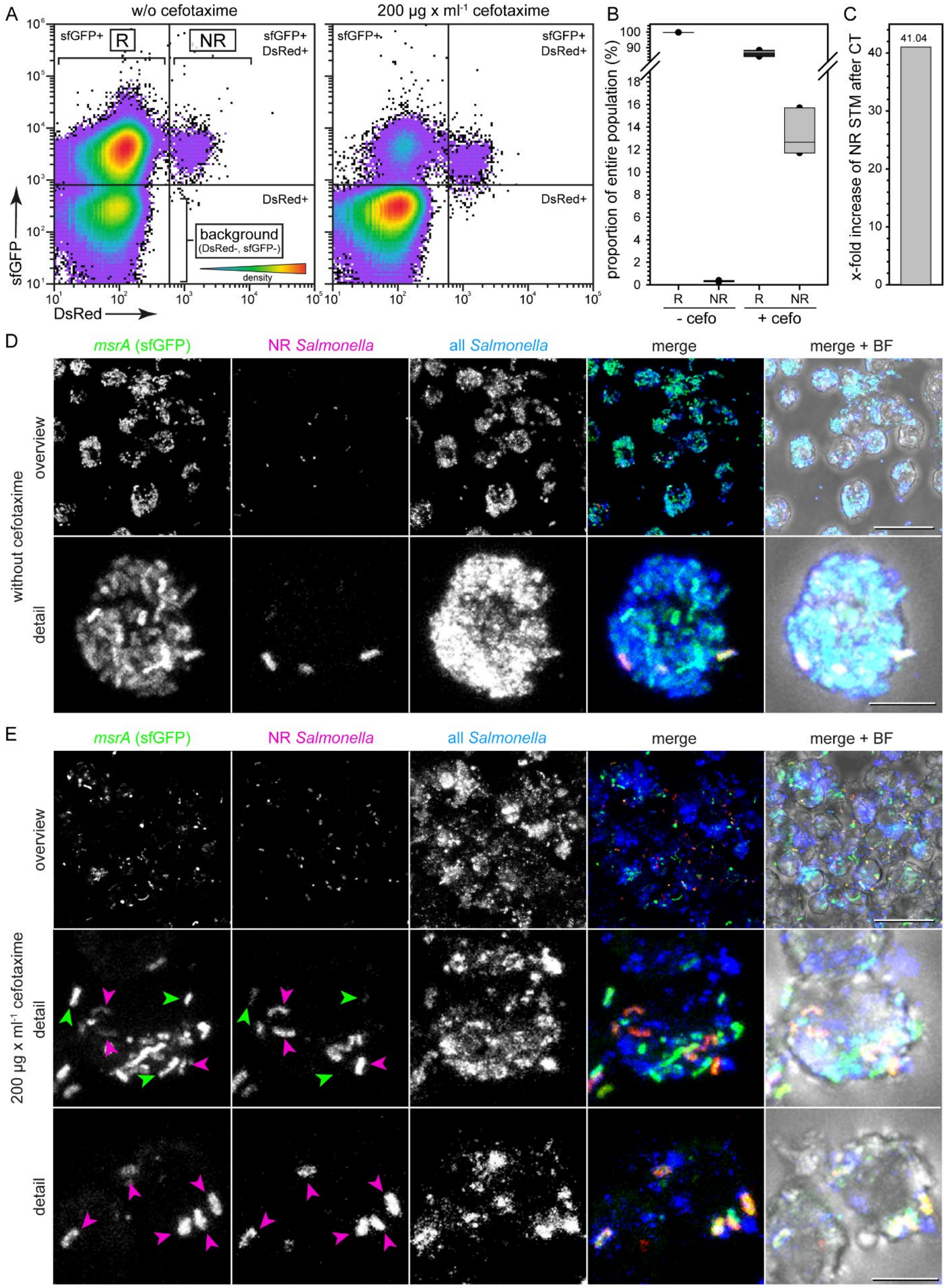

Supplementary Fig. S 6: Cefotaxime treatment increases intracellular population of non-replicating STM WT. STM WT harboring AHT-inducible (NR) dual-fluorescence reporter for *msrA* was grown o/n in LB medium in the presence of AHT. Before infection, AHT was removed. RAW264.7 macrophages were infected, cefotaxime was added 10 h p.i. to cells if indicated, and

cells were lysed at 24 h p.i. released STM were recovered, fixed and subjected to FC analyses. Alternatively, cells were fixed for fluorescence microscopy. A) Plotting of the entire intracellular bacterial population against their AHT-induced DsRed and P_{msrA}-induced sfGFP intensity shows a high reduction of R STM after antibiotic treatment. B) Quantification of the proportion of R and NR STM within the population with and without antibiotic treatment. C) Calculated x-fold increase of the proportion of NR compared to R STM after cefotaxime treatment (CT). Means and standard deviations of a representative experiment are shown. D, E) Microscopy of intracellular R and NR STM without (D), and with cefotaxime treatment (E). Fixed intracellular STM were immunostained against O antigen (blue). Induction of *msrA* is shown in green, red fluorescence (displayed magenta) is present or absent in NR STM or R STM, respectively. Merge overlays micrographs with and without overlay of brightfield (BF) channels are shown. NR STM and R STM in representative cells are indicated by magenta and green arrowheads, respectively. Scale bars, 20 µm (overview), 5 µm (detail).

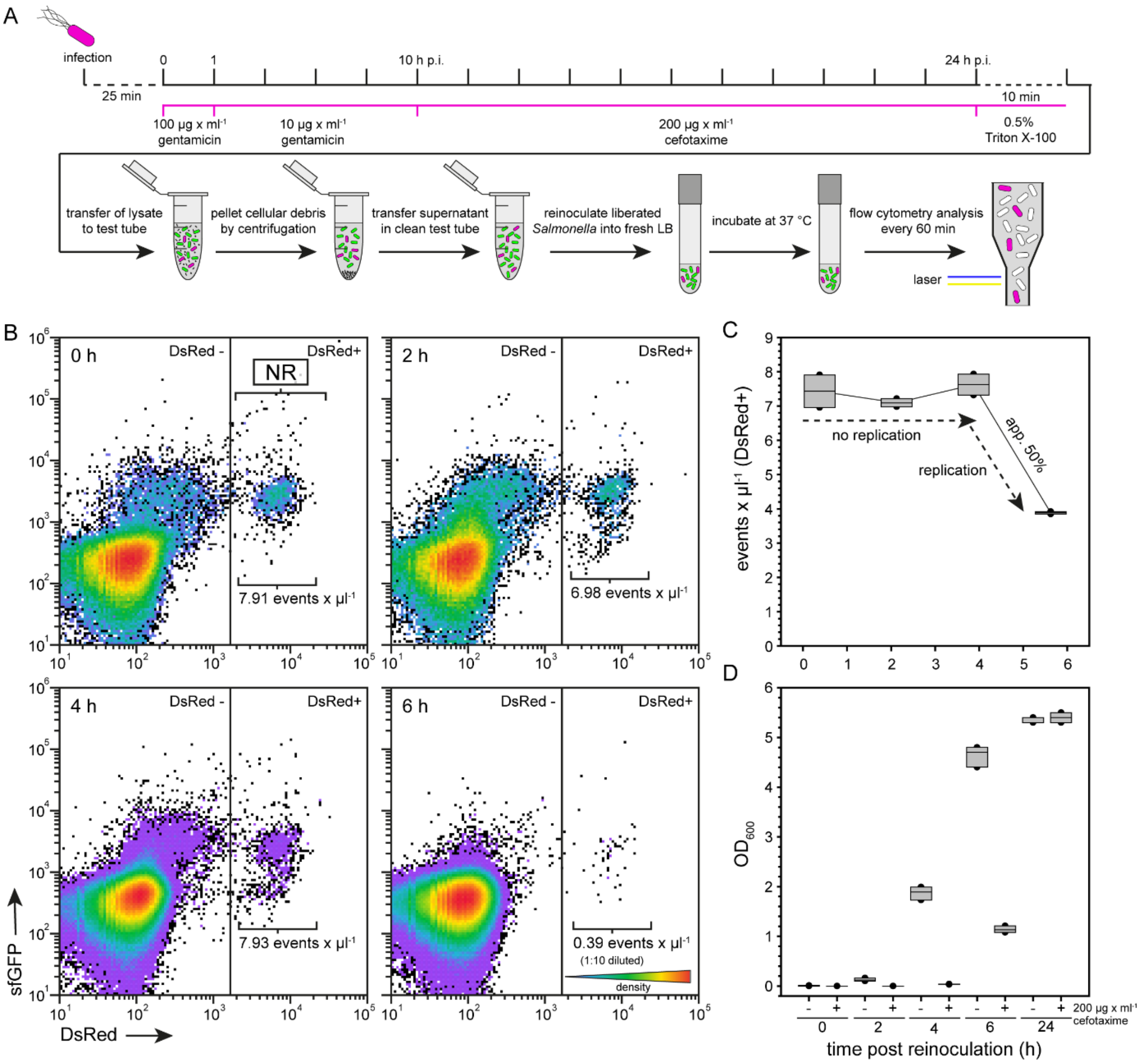

Supplementary Fig. S 7: Schematic overview of infection and lysis of host cells, reinoculation into fresh LB, and subsequent cytometric analysis. A) RAW264.7 macrophages were infected by STM harboring various reporters. After killing of non-phagocytosed STM by gentamicin, cell culture medium was replaced by medium containing 200 µg x ml^{-1} cefotaxime at 10 h p.i. After further incubation, host cells were lysed 24 h p.i. After removal of cell debris, liberated STM were reinoculated into fresh LB medium and incubated at 37 °C. At indicated time points samples were taken, fixed and subjected to FC. Quantification of DsRed-positive STM was performed using an Attune NxT cytometer. B) STM WT harboring AHT-inducible (NR) dual-fluorescence reporter for *msrA* was grown o/n in LB medium in the presence of AHT. Before infection, AHT was removed. RAW264.7 macrophages were infected, 10 h p.i. cefotaxime was added to the cells and then lysed 24 h p.i. Liberated STM were recovered, reinoculated into fresh LB and incubated at 37 °C. At indicated time points samples were taken, fixed and subjected to FC. C) The relative amount (events x µl^{-1}) of DsRed-positive NR STM in the culture was determined. A loss of DsRed-positive events over time indicates that NR STM are growth-competent and start to replicate. D) In addition, the

optical density was measured. Means and standard deviations of a representative experiment are shown.

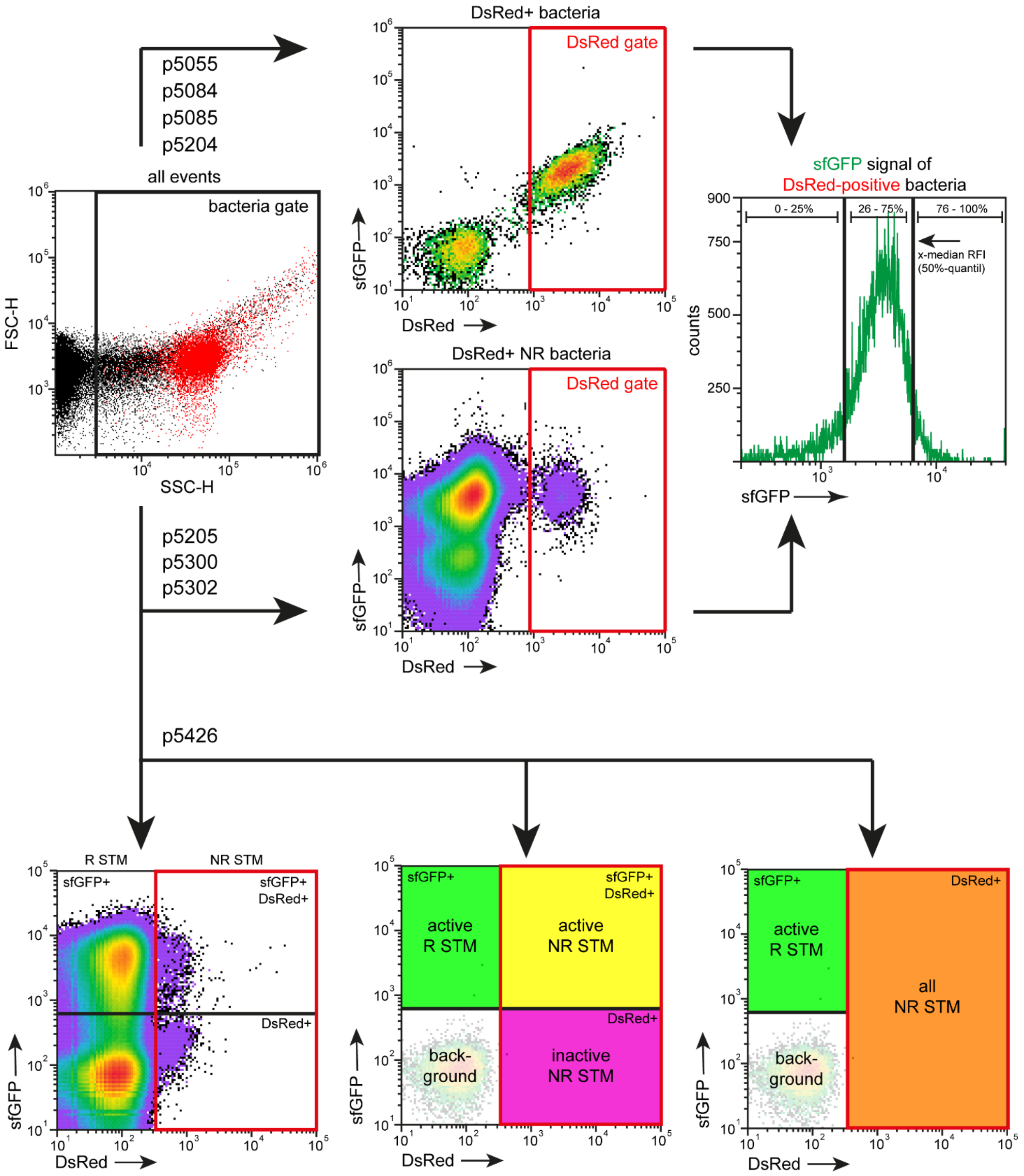

Supplementary Fig. S 8: Gating strategy for flow cytometry analyses. STM strains harbored following reporter plasmids: p5204 (P_{EM7}::*dsred* P_{cypD}::*sfgfp* frameshift), p5055 (P_{EM7}::*dsred* P_{htrA}::*sfgfp*), p5084 (P_{EM7}::*dsred* P_{msrA}::*sfgfp*), p5085 (P_{EM7}::*dsred* P_{trxA}::*sfgfp*) for constitutively expressed DsRed and stress-induced sfGFP. Reporter plasmids p5205 (P_{tetA}::*dsred* P_{msrA}::*sfgfp*), p5300 P_{tetA}::*dsred* P_{htrA}::*sfgfp*), p5302 (P_{tetA}::*dsred* P_{trxA}::*sfgfp*) for AHT-induced DsRed and stress-induced sfGFP. Reporter plasmids p5426 (P_{BAD}::*dsred* P_{tetA}::*sfgfp*) for arabinose-induced DsRed and AHT-induced sfGFP. Bacteria-sized particles were identified by SSC/FSC gates. For the gated events, DsRed fluorescence for STM with constitutive or inducible DsRed expression was used to gate

bacteria. For the DsRed-positive events, median values for sfGFP relative fluorescence intensities were determined (x-median RFI). For double-inducible reporter p5246, gating of sfGFP-positive, DsRed-positive, and double-positive STM are performed as indicated to distinguish active R STM, inactive NR STM, and active NR STM, respectively.

Supplementary References

1 Noster, J. *et al.* Proteomics of intracellular *Salmonella enterica* reveals roles of *Salmonella* pathogenicity island 2 in metabolism and antioxidant defense. *PLoS Pathog* **15**, e1007741, doi:10.1371/journal.ppat.1007741 (2019).

2 Bender, J. K., Wille, T., Blank, K., Lange, A. & Gerlach, R. G. LPS structure and PhoQ activity are important for Salmonella Typhimurium virulence in the Galleria mellonella infection model [corrected]. *PLoS One* **8**, e73287, doi:10.1371/journal.pone.0073287 (2013).

3 Schulte, M., Olschewski, K. & Hensel, M. Fluorescent protein-based reporters reveal stress response of intracellular *Salmonella enterica* at level of single bacterial cells. *Cell Microbiol*, doi:10.1111/cmi.13293 (2020).

4 Namakchian, M., Kassler, K., Sticht, H., Hensel, M. & Deiwick, J. Structure-based functional analysis of effector protein SifA in living cells reveals motifs important for *Salmonella* intracellular proliferation. *Int J Med Microbiol* **308**, 84-96, doi:10.1016/j.ijmm.2017.09.004 (2018).

5 Guzman, L. M., Belin, D., Carson, M. J. & Beckwith, J. Tight regulation, modulation, and high-level expression by vectors containing the arabinose PBAD promoter. *J Bacteriol* **177**, 4121-4130, doi:10.1128/jb.177.14.4121-4130.1995 (1995).
