# Supplementary material for: The protected physiological state of intracellular Salmonella enterica persisters reduces host cell-imposed stress
Source: Commun Biol. 2021 May 4;4:520. doi: 10.1038/s42003-021-02049-6 (PMC8096953; doi:10.1038/s42003-021-02049-6)
Supplement: Supplementary file 3 — Reporting Summary [file 42003_2021_2049_MOESM3_ESM.pdf]

nature research

Corresponding author(s): Michael Hensel

Last updated by author(s): Mar 15, 2021

# Reporting Summary

Nature Research wishes to improve the reproducibility of the work that we publish. This form provides structure for consistency and transparency in reporting. For further information on Nature Research policies, see our Editorial Policies and the Editorial Policy Checklist.

## Statistics

For all statistical analyses, confirm that the following items are present in the figure legend, table legend, main text, or Methods section.

| n/a | Confirmed | |
|---|---|---|
| ☐ | ☒ | The exact sample size (*n*) for each experimental group/condition, given as a discrete number and unit of measurement |
| ☐ | ☒ | A statement on whether measurements were taken from distinct samples or whether the same sample was measured repeatedly |
| ☐ | ☒ | The statistical test(s) used AND whether they are one- or two-sided<br>*Only common tests should be described solely by name; describe more complex techniques in the Methods section.* |
| ☐ | ☒ | A description of all covariates tested |
| ☐ | ☒ | A description of any assumptions or corrections, such as tests of normality and adjustment for multiple comparisons |
| ☐ | ☒ | A full description of the statistical parameters including central tendency (e.g. means) or other basic estimates (e.g. regression coefficient) AND variation (e.g. standard deviation) or associated estimates of uncertainty (e.g. confidence intervals) |
| ☐ | ☒ | For null hypothesis testing, the test statistic (e.g. *F*, *t*, *r*) with confidence intervals, effect sizes, degrees of freedom and *P* value noted<br>*Give P values as exact values whenever suitable.* |
| ☐ | ☒ | For Bayesian analysis, information on the choice of priors and Markov chain Monte Carlo settings |
| ☐ | ☒ | For hierarchical and complex designs, identification of the appropriate level for tests and full reporting of outcomes |
| ☐ | ☒ | Estimates of effect sizes (e.g. Cohen's *d*, Pearson's *r*), indicating how they were calculated |

*Our web collection on statistics for biologists contains articles on many of the points above.*

## Software and code

Policy information about availability of computer code

| | |
|---|---|
| Data collection | Flow cytometry data were collected and analysed by intrument specific software Attune NxT 3.12 or higher. Microscopy images were collected by Leica LAS software. |
| Data analysis | Not relevant. |

For manuscripts utilizing custom algorithms or software that are central to the research but not yet described in published literature, software must be made available to editors and reviewers. We strongly encourage code deposition in a community repository (e.g. GitHub). See the Nature Research guidelines for submitting code & software for further information.

## Data

Policy information about availability of data

All manuscripts must include a data availability statement. This statement should provide the following information, where applicable:

- Accession codes, unique identifiers, or web links for publicly available datasets
- A list of figures that have associated raw data
- A description of any restrictions on data availability

Source data underlying the graphs and micrographs shown in Fig, 2, Fig. 3, Fig. 4, Fig. 5, and Fig. 6 are available as Supplementary Data at the Zenodo repository: DOI: 10.5281/zenodo.4592976

# Field-specific reporting

Please select the one below that is the best fit for your research. If you are not sure, read the appropriate sections before making your selection.

[x] Life sciences    [ ] Behavioural & social sciences    [ ] Ecological, evolutionary & environmental sciences

For a reference copy of the document with all sections, see nature.com/documents/nr-reporting-summary-flat.pdf

# Life sciences study design

All studies must disclose on these points even when the disclosure is negative.

| | |
|---|---|
| Sample size | For cytometry, at least 10 000 DsRed-positive events were measured. |
| Data exclusions | Data exclusion was not performed and not relevant. |
| Replication | For each experiment, three technical replicated were measured, except of samples shown in Figure S1A, S1H and S5D. |
| Randomization | Randomization was not relevant. |
| Blinding | Blinding was not relevant. |

# Reporting for specific materials, systems and methods

We require information from authors about some types of materials, experimental systems and methods used in many studies. Here, indicate whether each material, system or method listed is relevant to your study. If you are not sure if a list item applies to your research, read the appropriate section before selecting a response.

## Materials & experimental systems

| n/a | Involved in the study | |
|---|---|---|
| [ ] | [x] | Antibodies |
| [ ] | [x] | Eukaryotic cell lines |
| [x] | [ ] | Palaeontology and archaeology |
| [x] | [ ] | Animals and other organisms |
| [x] | [ ] | Human research participants |
| [x] | [ ] | Clinical data |
| [x] | [ ] | Dual use research of concern |

## Methods

| n/a | Involved in the study | |
|---|---|---|
| [x] | [ ] | ChIP-seq |
| [ ] | [x] | Flow cytometry |
| [x] | [ ] | MRI-based neuroimaging |

## Antibodies

| | |
|---|---|
| Antibodies used | BD Difco rabbit anti Salmonella O antisera Lot: 0077470, Cy5- conjugated AffiniPure goat anti rabbit IgG (H+L) Jackson Immuno Research Lot: 62334 |
| Validation | Antibodies were used in various studies e.g. https://doi.org/10.1016/j.ijmm.2018.11.001 |

## Eukaryotic cell lines

Policy information about cell lines

| | |
|---|---|
| Cell line source(s) | RAW264.7 macrophages (American Type Culture Collection, ATCC no. TIB-71), RAW264.7 macrophages stably transfected with LAMP1-GFP were used |
| Authentication | Macrophage cell lines were authenticated by expression of surface markers, phagocytosis and generation of reactive oxygen species |
| Mycoplasma contamination | Cell lines were regularly screened for Mycoplasma contamination using a commercial test kit |
| Commonly misidentified lines (See ICLAC register) | Not relevant |

## Flow Cytometry

### Plots

Confirm that:

- [x] The axis labels state the marker and fluorochrome used (e.g. CD4-FITC).
- [x] The axis scales are clearly visible. Include numbers along axes only for bottom left plot of group (a 'group' is an analysis of identical markers).
- [x] All plots are contour plots with outliers or pseudocolor plots.
- [x] A numerical value for number of cells or percentage (with statistics) is provided.

### Methodology

| | |
|---|---|
| Sample preparation | Sample preparation is decribed in the Material and Method section of this manuscript in detail |
| Instrument | Attune NxT (ThermoFisher Scientific) |
| Software | Attune NxT (ThermoFisher Scientific) version 3.12 or higher |
| Cell population abundance | Not relevant |
| Gating strategy | The gating strategy is described Material and Methods and a figure exemplifying the gating strategy is shown as Supplementary Fig. S8 |

- [x] Tick this box to confirm that a figure exemplifying the gating strategy is provided in the Supplementary Information.
